# A new species of *Peucoglyphus* Bernhauer from New Guinea (Coleoptera, Staphylinidae, Staphylininae)

**DOI:** 10.3897/zookeys.210.3241

**Published:** 2012-07-24

**Authors:** Alexey Solodovnikov

**Affiliations:** 1 Zoological Museum, Natural History Museum of Denmark, Universitetsparken 15, Copenhagen 2100, Denmark

**Keywords:** Staphylinini, Philonthina *Peucoglyphus*, new species, New Guinea

## Abstract

*Peucoglyphus ken* **sp. n.**, a new species from New Guinea is described. Adding the new species, this rare Wallacean genus from the tribe Staphylinini (subtribe Philonthina) currently includes five species. An updated identification key for the genus is provided.

## Introduction

*Peucoglyphus* Bernhauer, 1926 is a genus of the rove beetle tribe Staphylinini (subtribe Philonthina) that was enacted for *Peucoglyphus corporaali* Bernhauer, 1926, a species from Buru Island in Indonesia (Bernhauer 1926). Since then nothing at all had been published about *Peucoglyphus* for almost a century until Schillhammer (2011) added three more species: *Peucoglyphus solomonicus* Schillhammer, 2011 from Solomon Islands, *Peucoglyphus celebensis* Schillhammer, 2011 from the island of Sulawesi (Indonesia), and *Peucoglyphus balkei* Schillhammer, 2011 from Irian Jaya. The abovementioned paper also provided an updated diagnosis and notes on the phylogenetic affinities of that rare genus. It immediately allowed me to identify a puzzling specimen from New Guinea that I had on loan from the Netherlands Centre for Biodiversity (Naturalis) in Leiden, as a new species of *Peucoglyphus*. Here I provide the description of this new species along with some comparative notes, and accordingly update the identification key to species of *Peucoglyphus* of Schillhammer (2011).

## Material and methods

The holotype of the new species is kept at the Netherlands Centre for Biodiversity (Naturalis) at Leiden (NCBN, M.E. Gassó Miracle and A. van Assen). All photographs illustrating the description were taken by Ken Puliafico (Copenhagen) with a Leica DFC 420 camera attached to a Leica MZ16A microscope with the help of Leica Application Suite (Leica Microsystems, 2003-2007) and Automontage Pro (Synoptics Ltd, 1997–2004).

### Peucoglyphus ken

sp. n.

urn:lsid:zoobank.org:act:DCA914C6-A2E4-4EC1-9E89-7EFBAFD31FBD

http://species-id.net/wiki/Peucoglyphus_ken

Figs 1–5

#### Type material examined.

**Indonesia** (West Papua): Holotype [with antennomeres 4–11 and labial palps missing], male, “Neth. Ind. - Amer. New Guinea Exp. 1938 Lake Habbema, 3250–3300 m, Ult. VII-ult. VIII L.J. Toxopeus leg.”/ Defective *Mysolius*? [handwritten label in red ink]/ sec. M. Cameron”/ Holotype *Peucoglyphus ken* sp. n. A. Solodovnikov det. 2012” [here the labels are quoted verbatim, individual labels separated by a slash] (NCBN).

#### Description.

**Figures 1–5.**
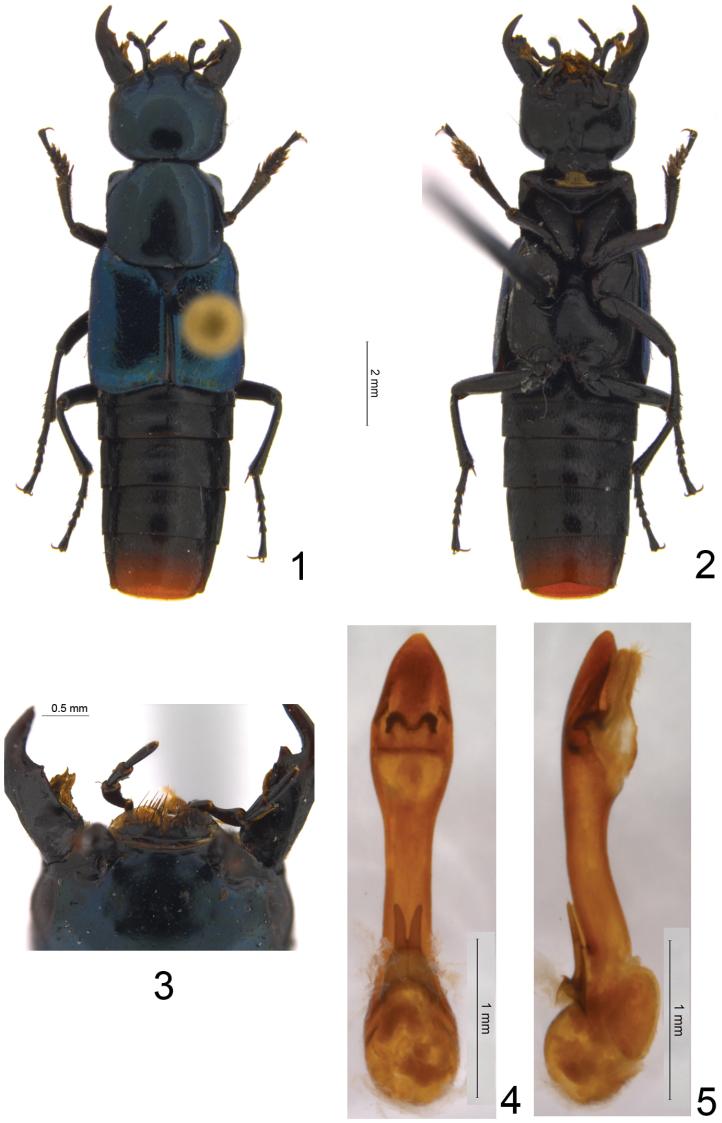
*Peucoglyphus ken* sp. n.: **1** habitus **2** body in ventral view **3** anterior portion of head **4** aedeagus in parameral view **5** aedeagus in lateral view.

15.5 mm long (measured from apex of opened mandibles to apex of abdomen). Habitus: Figs 1, 2. Black and shiny, head and pronotum with deep dark blue metallic glance, elytra brilliant glossy, with strong metallic blue glance, scutellum brilliant, but darker, with violet glance; mouthparts dark brown to black; legs black, except femora at base dark brown; apex of abdomen beginning from segment VIII reddish-brown.

Head large, with rounded posterior angles, only slightly wider than long (head length from base of labrum to neck 2.5 mm; maximal head width, at eyes 2.7 mm); tempora 1.8 times as long as eyes, eyes posteriorly shifted dorsad; surface of head smooth with micropunctation faint and sparse at disk, but coarser and denser at tempora; frons with one pair of large setiferous punctures, each located near anterior part of internal margin of eye; other large, possibly setiferous punctures arranged in irregular groups behind eye and along posterior margin of head; tempora with one large setiferous puncture located closer to posterior margin of head than to posterior margin of eye; bilobed labrum with semi-membranous yellowish extension developed along its entire apical margin (Fig. 3). Pronotum slightly transverse (length along midline 2.5 mm, maximal width 2.8 mm), with parallel lateral sides, broadly rounded posterior angles and distinct anterior angles; at sides slightly sinuate in front of base and just posterior to anterior angles; micropunctation as on disk of head: very sparse and faint; large possibly setiferous punctures are grouped at anterior corners, 2–3 on disk on each side and some along posterior margin. Elytra wider and longer than pronotum (elytral length from base to apical margin 3.5 mm, maximal elytral width 3.7 mm), their surface with faint and sparse micropunctation and dense microsculpture, slightly longitudinally wrinkled at base and along apical margin; each elytron laterally without carina; scutellum faintly punctate. Metaventrite without conspicuous fold posterolaterally (illustrated in Schillhammer 2011 for *Peucoglyphus balkei* in fig. 4). Abdomen: first five visible tergites (III–VII) medially more or less smooth, impunctate, but laterally and basally with more or less coarse punctuation; all tergites with only one basal carina, tergites IV–VI with more or less deep transversal impression; male sternite VIII with medio-apical emargination; male sternite IX with short slightly asymmetrical poorly sclerotised basal portion, and with slightly bilobed apex.

Aedeagus in parameral view (Fig. 4) with median lobe having massive apical portion that is as wide as basal bulb, in lateral view (Fig. 5) slightly curved, with very short paramere consisting of two symmetrical lobes.

#### Bionomics and distribution.

Known from the type locality only. No data about the collecting method or bionomics of the holotype is available.

#### Etymology.

With pleasure I dedicate the new species to Kenneth (Ken) Puliafico, currently a digitalization assistant at the Department of Entomology at the Natural History Museum of Denmark. Ken’s excellent work as a specimen photographer and database specialist, aiming to digitize thousands of Coleoptera types kept in our collection, is a notable contribution towards the better infrastructure for beetle systematics. The species name “ken” is a noun in apposition.

#### Comparison.

Based on the rather small eyes that are shorter than tempora (Fig. 1), the dark legs (Figs 1 and 2), and the distinct nuchal constriction (Fig. 1), *Peucoglyphus ken* can be placed near *Peucoglyphus solomonicus* Schillhammer, 2011, at least diagnostically. However, *Peucoglyphus ken* differs from *Peucoglyphus solomonicus* in proportions of the forebody (cf. Fig 1 and fig. 2 in Schillhammer 2011), in the color of the apex of the abdomen which is reddish brown, in lacking an arcuate row of large setiferous punctures extending from infraorbital area on to tempora, and in the structure of the abdominal tergites having only one basal carina (contrary to two carinae in *Peucoglyphus solomonicus*). Also, from all other congeners with known males *Peucoglyphus ken* strikingly differs in the shape of the aedeagus (cf. Figs 4, 5 and fig. 11 in Schillhammer 2011).

#### Remarks.

The new species matches the generic diagnosis of *Peucoglyphus* provided in Schillhammer (2011) in all characters except lacking temporal carina (formed by confluent punctural grooves) and except slightly different configuration of the semi-membranous extension of labrum. Temporal carina is present in all other species of the genus, and the semi-membranous extension of labrum is developed along the entire width of labral lobes in *Peucoglyphus ken* (Fig. 3), but laterally reduced in all other species of the genus. However, the structure of the aedeagus in *Peucoglyphus ken* is remarkably different from all other species of *Peucoglyphus* with known males. Unlike *Peucoglyphus corporaali*, *Peucoglyphus balkei* and *Peucoglyphus solomonicus*, the aedeagus of *Peucoglyphus ken* has a distinct but strongly reduced paramere, and enlarged (in dorsal or ventral view, Fig. 4) apical portion of the median lobe without the subapical tooth so characteristic for other species of *Peucoglyphus* (cf. Fig. 5 and figs 10–12 in Schillhammer 2011). The shape of the paramere in *Peucoglyphus ken* suggests that in other species of the genus it is even stronger reduced, rather than fused to the median lobe, the condition earlier not clearly understood (Schillhammer 2011). Since the antennae and labial palps are largely missing in the holotype of *Peucoglyphus ken*, the corresponding structures cannot be compared with other congeners. Noteworthy, that the laterally reduced semi-membranous extension of the labrum is among the characters that distinguish *Peucoglyphus* from the closely related genera of Philonthina: *Leucitus* Fauvel, 1878, *Actinus* Fauvel, 1878 and *Mysolius* Fauvel, 1878, all having such extension fully developed. The fully developed semi-membranous extension of labrum in *Peucoglyphus ken* shared with them, and the structure of its aedeagus that is also rather similar to some species in those genera, confirm the affinities of *Peucoglyphus* noted in Schillhammer (2011).

##### Key to species of *Peucoglyphus* (after Schillhammer 2011, modified to include *Peucoglyphus ken*)

**Table:** 

1	Eyes small, markedly shorter than tempora	2
–	Eyes large, slightly to distinctly longer than tempora	4
2	Fore legs and mesofemora black or at least very dark brown; nuchal ridge sharp throughout its entire length, nuchal constriction distinct	3
–	Fore legs and mesofemora reddish; nuchal ridge convex, rather fine, almost obsolete in middle, nuchal constriction indistinct	*Peucoglyphus corporaali*
3	Semi-membranous extension of labrum developed along median part of labral lobes only, laterally reduced; temporal carina formed by confluent punctural grooves present	*Peucoglyphus solomonicus*
–	Semi-membranous extension of labrum developed along the entire width of labral lobes; temporal carina formed by confluent punctural grooves absent	*Peucoglyphus ken*
4	Legs entirely bright reddish.	*Peucoglyphus balkei*
–	Legs black, pro- and mesofemora bright reddish	*Peucoglyphus celebensis*

## Supplementary Material

XML Treatment for Peucoglyphus ken
